# Synergistic Insecticidal Activity Against *Hyphantria cunea* by Cry9Aa3 Mutants and Cry1Ah Combinations

**DOI:** 10.3390/ijms26083497

**Published:** 2025-04-08

**Authors:** Pengdan Xu, Zeyu Wang, Ying Zhang, Jiaxing Han, Changlong Shu, Min Liao, Jie Zhang, Lili Geng

**Affiliations:** 1 School of Plant Protection, Anhui Agricultural University, Hefei 230026, China; 2 State Key Laboratory for Biology of Plant Diseases and Insect Pests, Institute of Plant Protection, Chinese Academy of Agricultural Sciences, Beijing 100193, China

**Keywords:** *Hyphantria cunea*, *Bacillus thuringiensis*, *cry9*-type genes, mutant, synergistic effect

## Abstract

The larvae of *Hyphantria cunea* feed on plant leaves, causing significant losses to forestry and agricultural production. At present, *cry1* genes such as *cry1Ac* and *cry1Ah* are mainly used to control *H. cunea*. To delay the problem of pest resistance induced by a single insecticidal gene, it is crucial to discover and develop new insecticidal genes or gene combinations. This study found *cry9Aa3* and *cry9Aa4* showed insecticidal activity against *H. cunea*. The toxicity of 14 mutants of Cry9Aa3 was analyzed and the LC_50_ of the triple-amino-acid substitution mutant 316LRG318AAA was 3.69 μg/g, which represents a 1.49-fold increase in insecticidal activity compared to Cry9Aa3. Additionally, enhanced stability of this mutant was detected in the midgut juice of *H. cunea*. Cry9Aa3 and 316LRG318AAA, in combination with Cry1Ah, demonstrated synergistic effects against *H. cunea*, with synergistic factors of 4.76 and 8.33, respectively. This study has identified the mutant 316LRG318AAA and its combination with Cry1Ah as exhibiting high toxicity against *H. cunea*, providing valuable genetic resources for the development of transgenic poplars and holding significant importance for delaying resistance in this pest.

## 1. Introduction

*Hyphantria cunea* belongs to the order Lepidoptera, family Arctiidae [1]. Originally found in North America, it spread through Asia and Europe after being introduced to East Asia in the early 1940s [2]. *H. cunea* was first discovered in 1979 in the Dandong region of Liaoning Province, China [3]. It is listed as a quarantine object by both the Ministry of Agriculture and Rural Affairs of the People’s Republic of China and the National Forestry and Grassland Administration [4,5]. *H. cunea* is a voracious polyphagous herbivore, and its hosts include more than 600 species, including forests, fruit trees, and various crops [6,7,8,9]. It has a strong adaptability to new hosts, which enables it to breed quickly in the invasion area [5]. During the past decades, the spread of *H. cunea* has accelerated dramatically, causing enormous economic losses to the forestry sector in China [10]. Common control measures for *H. cunea* include chemical control, physical control, and biological control. Chemical pesticides offer the advantage of rapid effectiveness, but the long-term use poses concerns over the 3R issues (resistance, resurgence, and residue) [11]. Physical control primarily involves manual removal by labor or the use of mechanical equipment, but these methods are not suitable for widespread application or large-scale control [12]. Biological control mainly utilizes *Bacillus thuringiensis* (Bt), *Beauveria bassiana*, and *H. cunea* nuclear polyhedrosis virus (HcNPV) [13,14,15]. Among these, Bt is a promising option due to its high toxicity, and it can be used in conventional spray formulations [16,17].

Bt, a group of pathogenic bacteria toxic to insects, provides a valuable resource due to its ability to synthesize insecticidal proteins [18]. During the formation of spores, it produces insecticidal crystal proteins (Cry). Cry proteins have played a significant role in controlling various pests, including Lepidoptera [19,20], Diptera [21], Coleoptera [22], and Hemiptera [23]. When ingested by insect larvae, the protoxin is activated by midgut proteases. These activated toxins then bind to specific protein receptors in the midgut epithelium, causing perforation of the midgut epithelial cells and ultimately leading to death [24]. Gene mutants are the primary driver for the functional improvement of Bt toxins. Functional evolutionary mutants in Bt toxins can occur spontaneously under natural conditions [25]. However, deliberate genetic engineering of Bt genes is currently the most significant means to increase their insecticidal activity. Mutants include rational design based on the structure of Bt proteins and random mutants performed when the structure–function relationship is unknown. Specifically, site-directed mutagenesis targeting the α-region of the helix structure in Domain I, the loop structure in Domain II, and the β-sheet structure in Domain III, which are key functional regions, can be used to design mutants with improved insecticidal activity [26,27,28].

Currently, *cry1* genes such as *cry1Ab* [29] and *cry1Ah* [30] have been found to exhibit insecticidal activity against *H. cunea*. The Cry1Ab protein exerts its insecticidal function by binding to Aminopeptidase N (APN) in the brush border membrane vesicles (BBMVs) of *H. cunea*’s midgut, with a median lethal concentration (LC_50_) of 4.34 μg/mL. At a concentration of 10 μg/mL, the Cry1Ah protein has a 100% mortality against larvae of the *H. cunea* [30]. McCown et al. introduced Bt *crylAa* gene into *Populus*, obtaining a transgenic plant with a mortality of 60% against *Malacosoma neustria* [31]. China’s transgenic technology has also developed rapidly. In 1991, Wu et al. introduced the Bt gene into European black poplar [32]. In 1993, China reported on transgenic black poplar with the *cry1Ac* gene, which increased the mortality of the Lepidopteran pest *Lymantria dispar* by over 80% compared to conventional poplar [33]. Transgenic poplar with the *cry1Ah* gene has shown significant effects in controlling *H. cunea* [30], and researchers found that Bt transgenic crops did not affect the community structure of species [34].

Currently, the genes reported to have good insecticidal effects against *H. cunea* are mainly *cry1*-type genes. To prevent insect resistance, it is particularly important to search for new *cry* genes or mutants with insecticidal activity against *H. cunea*. In 1991, the first *cry9Aa* gene was cloned, and currently, five *cry9A*-type genes have been cloned. Cry9A-type proteins exhibit insecticidal activity against many Lepidopteran pests. The Cry9Aa2 protein shows insecticidal activity against *Phthorimaea operculella* [35]. Cry9Aa3 has an LC_50_ of 0.70 μg/g against *Chilo suppressalis* larvae [36], and Cry9Aa5 is poisonous against *Spodoptera exigua* [37]. Notably, *cry9*-type genes do not exhibit cross-resistance with *cry1*-type genes. Furthermore, Cry9Ca1 and Cry1Ab5 bind to distinct sites within the BBMVs of *Ostrinia nubilalis* [38]. In both *C. suppressalis* and *Scirpophaga incertulas*, there is no observed competition for receptor sites between Cry1Ac and Cry9C [39]. In this study, we analyzed the insecticidal activity of Cry9A-type proteins against *H. cunea* and screened for Cry9Aa3 mutants with increased insecticidal activity, which involves mutating three amino acids in the Loop region of Domain II. We further analyzed the reasons for the increased insecticidal activity of the mutants. At the same time, it is found that this mutant has a synergistic effect with the Cry1Ah protein. Novel Bt genes, mutant, and gene combinations with high toxicity against *H. cunea* have been obtained in this research, providing a genetic resource for the creation of transgenic poplar, and being of great significance for delaying the resistance of *H. cunea*.

## 2. Results

### 2.1. Cry9Aa Protein Has Insecticidal Activity Against H. cunea

**Figure 1:**
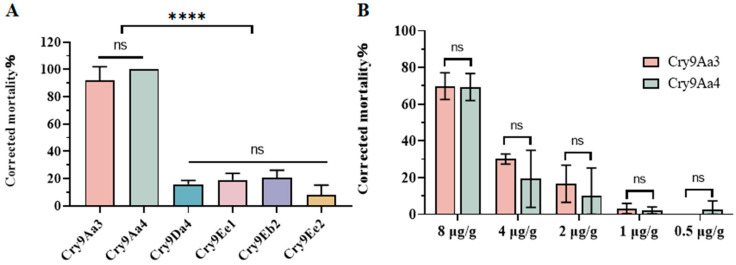
Insecticidal activity of Cry9 against *H. cunea*. (**A**) Bioassay analysis of Cry9-type proteins against *H. cunea*. (**B**) Bioassay analysis of Cry9Aa3 and Cry9Aa4 against *H. cunea*. The data in the figures were corrected mortality rates, with statistical significance indicated by asterisks (**** *p* < 0.0001, ns represents no significant difference, three biological replicates for each treatment, with error bars indicating standard deviation). The original data were presented in Tables S2 and S3.

**Figure 2:**
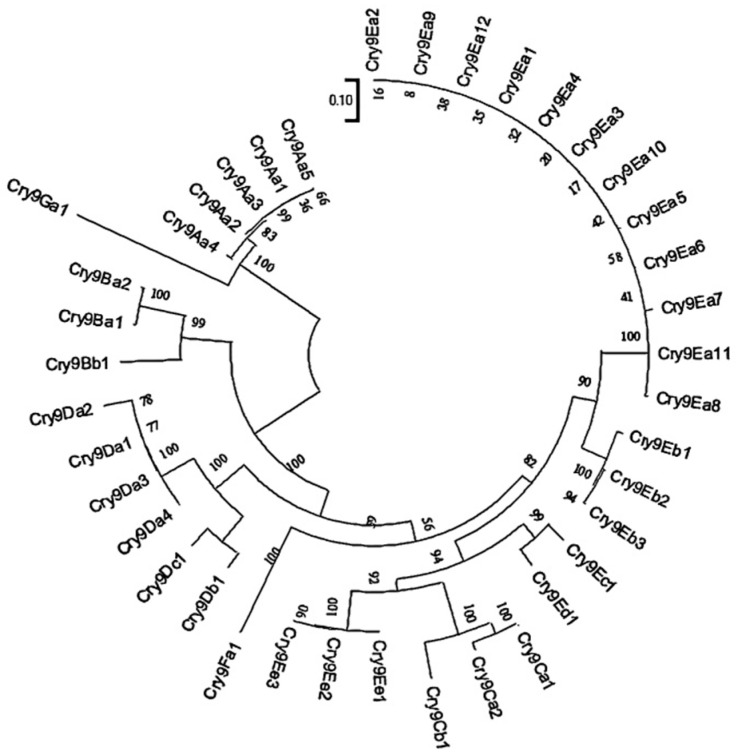
Phylogenetic tree of *cry9* genes.

Currently, 40 *cry9*-type genes have been cloned, among which Cry9A, Cry9B, Cry9C, Cry9E, Cry9F, and Cry9G proteins exhibit insecticidal activity against Lepidopteran pests (Table S1, Data source: Bacterial Pesticidal Protein Database, BPPRC). To verify the insecticidal activity of Cry9-type proteins against *H. cunea*, Cry9Aa3, Cry9Aa4, Cry9Da4, Cry9Eb2, Cry9Ee1, and Cry9Ee2 were extracted. Except for the truncated Cry9Aa3 protein, which has a size of 70 kDa, the other proteins are all around 130 kDa. When the protein concentration was 10 μg/g, it was found that the 4-day corrected mortality rates of *H. cunea* caused by Cry9Aa3 and Cry9Aa4 were 92.06% and 100.00%, respectively, while the insecticidal activities of Cry9Da4, Cry9Eb2, Cry9Ee1, and Cry9Ee2 proteins against *H. cunea* were all below 20% (Figure 1A). The insecticidal activities of Cry9Aa3 and Cry9Aa4 against *H. cunea* were further compared. At a protein concentration of 8 μg/g, they both exhibited high insecticidal toxicity against *H. cunea* larvae, with corrected mortality rates of 69.84% and 69.36%, respectively, showing no significant difference. When protein concentrations were 4 μg/g, 2 μg/g, 1 μg/g, and 0.5 μg/g, no significant difference was observed in the insecticidal activity against *H. cunea* of the two proteins (Figure 1B, Table S2). Upon constructing a phylogenetic tree for the *cry9* genes, it was found that *cry9Aa3* and *cry9Aa4* exhibit a very close phylogenetic relationship (Figure 2, Table S3), which might be one of the reasons for their similar insecticidal activities. The two genes exhibit 98% overall sequence identity at the amino acid level, with their N-terminal regions encoding functional domains I–III (residues 57–655) showing 99.67% sequence conservation.

### 2.2. Mutant of Cry9Aa3 Increased Insecticidal Activity Against H. cunea

**Figure 3:**
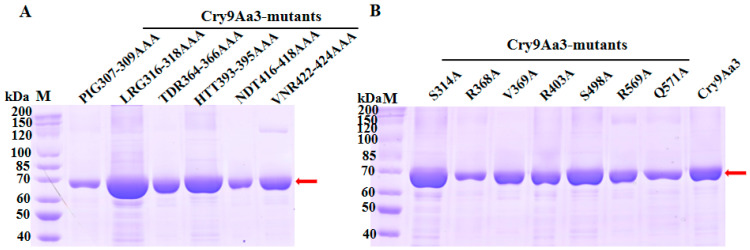
SDS-PAGE analysis of the extraction of Cry9Aa and its mutants. (**A**) SDS-PAGE analysis of the expression of Cry9Aa3 and its triple mutant proteins. The arrow indicates that the target proteins, with a molecular weight of 70 kDa, were detected. (**B**) SDS-PAGE analysis of purified Cry9Aa3 and its single mutant.

In total, 14 mutants were constructed through site-directed mutagenesis in our lab, including 8 single-point mutants and 6 triple-site mutations, which were mainly located in Domain II and Domain III [40]. The heterologously expressed Cry9Aa3 and its mutant proteins had a molecular weight of approximately 70 kDa (Figure 3A), consistent with the expected molecular weight. Cry9Aa3 and mutant proteins were purified using Ni^2+^-affinity chromatography to above 80% purity (Figure 3B).

**Figure 4:**
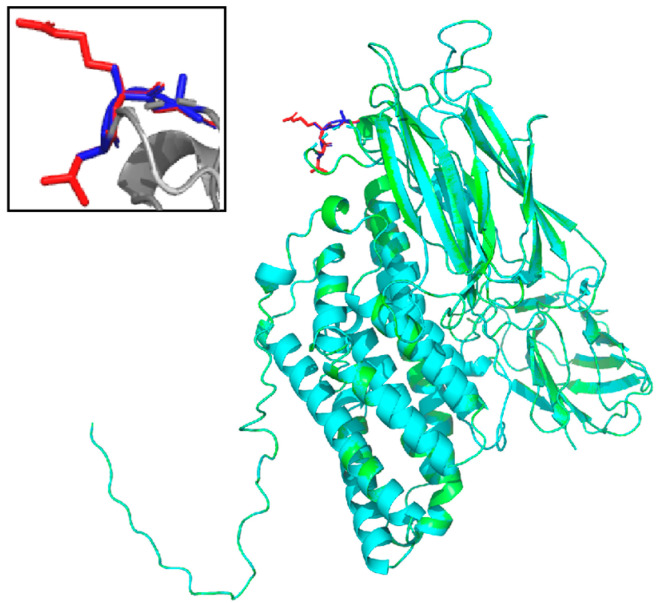
Cry9Aa3 model structure compared with 316LRG318AAA. Note: the Cry9Aa3 protein sequence is marked in green, and the 316LRG318AAA protein sequence is marked in cyan. Red represents the amino acid structure at positions 316–318 before mutation, and blue represents the amino acid structure after mutation. RMSD = 0.000. The structures of the Cry9Aa3 proteins were predicted and modeled by SWISS-MODEL. The rmsd values were calculated by aligning to the molecule using PyMOL software v2.5.0.

**Figure 5:**
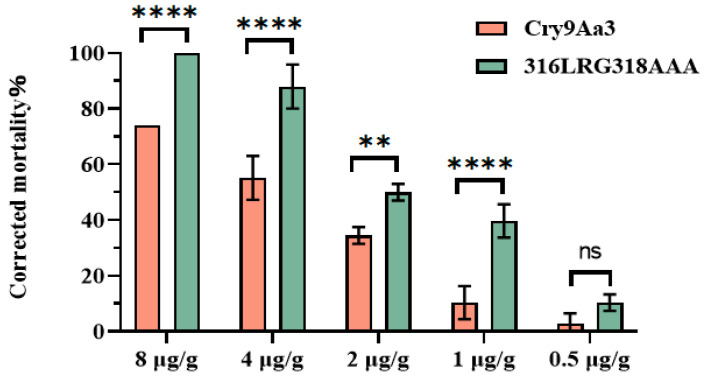
Insecticidal activity of Cry9Aa and 316LRG318AAA against *H. cunea*. Note: ** indicate *p* < 0.01, **** indicate *p* < 0.0001, three biological replicates for each treatment, with error bars indicating standard deviation. The original data were presented in Table S5.

Insecticidal activity of Cry9Aa3 and its mutant proteins against *H. cunea* larvae were analyzed and revealed that the 307PIG309AA and 416NDT418AAA mutants exhibited reduced insecticidal activity, while the 316LRG318AAA mutant showed increased insecticidal activity (Table 1). When the concentration of the 316LRG318AAA mutant was 5 μg/g, the corrected mortality against *H. cunea* was 76.92%, and at a concentration of 10 μg/g, the corrected mortality reached 100% (Table S4). Insecticidal activities of the remaining mutants did not significantly change. A three-dimensional structure simulation of Cry9Aa3 and 316LRG318AAA proteins was performed, and mutations were located on Loopα8 in Domain II (Figure 4). Further comparison between the 316LRG318AAA mutant and the wild-type Cry9Aa3 protein demonstrated that the corrected mortality with the mutant was significantly higher than Cry9Aa3 protein at concentrations of 1, 2, 4, and 8 μg/g (Figure 5, Table S5).

### 2.3. Enhanced Insecticidal Activity of Cry9Aa3 Mutant Is Related to Higher Stability in Midgut Juice

**Figure 6:**
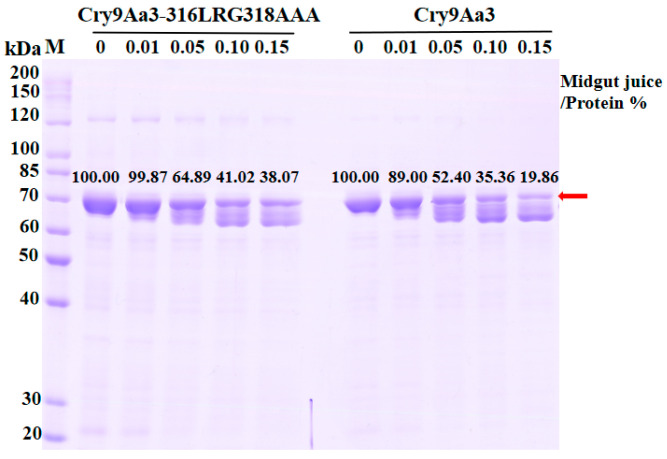
SDS-PAGE analysis of the stability of Cry9Aa3 and 316LRG318AAA in the midgut juice of *H. cunea*. The upper bands represent the inactive precursor protein fragment. The lower bands correspond to the activated protein fragments. The red arrow and numerical labels are included to emphasize the stable presence of the unprocessed precursor.

We used the extracted midgut fluid of *H. cunea* to analyze the stability of wild-type Cry9Aa3 and mutant 316LRG318AAA. When the percentage of midgut juice to protein volume was 0.01%, 0.05%, 0.10%, and 0.15%, the proportions of inactivated mutant 316LRG318AAA fragments relative to the original protein were 99.87%, 64.89%, 41.02%, and 38.07%, respectively. For Cry9Aa3, the proportions of inactivated fragments were 89.00%, 52.40%, 35.36%, and 19.86%, respectively. The results indicated that the mutant protein is more stable in midgut juice (Figure 6), which is consistent with the finding that mutant 316LRG318AAA exhibits better insecticidal activity against *H. cunea*. This also suggests that the increased insecticidal activity of mutant 316LRG318AAA is related to its improved stability in the midgut juice of *H. cunea*.

### 2.4. No Significant Difference in the Binding Affinity of Cry9Aa3 and 316LRG318AAA to the BBMVs of H. cunea

**Figure 7:**
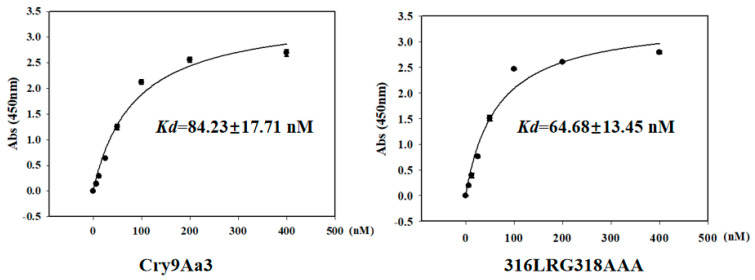
Binding assay of Cry9Aa3 and 316LRG318AAA to BBMVs of *H. cunea*. Saturation binding of biotinylated Cry9Aa3 and its mutant proteins bound to BBMVs of *H. cunea*. Error bars represent SD. Abs, absorbance. The original data were presented in Table S6.

The interaction between Bt proteins and midgut BBMVs is considered a crucial step for exerting insecticidal activity against target pests. Cry9Aa3 and its mutant was labelled by biotin (Figure S1), and we preliminarily investigated the binding of Cry9Aa3 and the mutant protein to BBMVs. The APN activity of the extracted BBMVs was 3.2 times that of the initial homogenate, indicating successful extraction of BBMVs. The affinity constants for the binding of BBMVs to Cry9Aa3 and 316LRG318AAA were 84.23 ± 17.71 (nM) and 64.68 ± 13.45 (nM), respectively (Figure 7, Table S6). These findings suggest that mutant 316LRG318AAA exhibits no significant difference in binding affinity to BBMVs of *H. cunea* compared to Cry9Aa3.

### 2.5. Cry9Aa3 and Mutant 316LRG318AAA Exhibit Synergistic Effects with Cry1Ah

The Cry1Ah protein exhibits insecticidal activity against *H. cunea* [30]. We analyzed whether Cry9Aa3 and its mutant exhibit synergistic effects with Cry1Ah. Bioassays were conducted on larvae of *H. cunea* to determine the LC_50_ of these three proteins. The LC_50_ values of *H. cunea* were calculated using SPSS software (V27.0), and the LC_50_ values for the combinations of Cry9Aa3 with Cry1Ah (1:1) and 316LRG318AA with Cry1Ah (1:1) were calculated using the formula mentioned in the bioassay methods. Simultaneously, observed LC_50_ values were also determined (Table 2). The bioassay results showed that the LC_50_ of the mutant 316LRG318AA for *H. cunea* was 3.48 μg/g, while the LC_50_ of Cry9Aa3 was 5.48 μg/g. Both of the measured LC_50_ values for the combinations of Cry9Aa3 and Cry1Ah (1:1) and 316LRG318AA and Cry1Ah (1:1) were lower than the expected LC_50_ values. The calculated synergism factors were 4.76 for Cry9Aa3 and Cry1Ah (1:1) and 8.33 for 316LRG318AA and Cry1Ah (1:1). Compared to Cry9Aa3, the insecticidal activity of 316LRG318AAA was 1.49 times higher, and the synergistic effect of the mutant with Cry1Ah was also higher than that of Cry9Aa3 with Cry1Ah.

## 3. Discussion

With the development of transgenic plants, researchers have introduced the *cry genes* from Bt, which exhibit high insecticidal activity against pests, into crops to achieve pest resistance and ensure crop yields. However, with the promotion and large-scale cultivation of transgenic crops, some pests have developed resistance to transgenic Bt crops due to selective pressure, posing a serious threat to the sustainable application of Bt products [41]. To address this issue, there is an urgent need to discover new insecticidal genes, modify target genes, and construct transgenic crops with stacked genes. Employing bivalent or multivalent transgenic insect-resistant crops, leveraging synergistic interactions between RNAi and Bt toxins, regularly renewing crop varieties, and conducting pest resistance monitoring are all essential strategies to delay the evolution of pest resistance [42,43,44,45]. Site-directed modification of target genes may not only enhance insecticidal activity against target insects, but also effectively delay the emergence of pest resistance. Simultaneously, screening for gene combinations with synergistic effects is also one of the important ways to delay pest resistance.

The proteins that exhibit insecticidal activity against *H. cunea* are mainly those of the Cry1-type proteins. As there are currently no reports of resistance to *cry9* genes, and Cry9A exhibits synergistic effects with Cry1-type proteins and Vip3A [46,47,48], they hold promising application prospects. In this study, we screened and obtained Cry9Aa3 and Cry9Aa4, which exhibit high toxicity against *H. cunea*. A phylogenetic tree was constructed for the *cry9* genes, which shows that *cry9Aa3* and *cry9Aa4* are closely related in phylogenetic evolution; this is consistent with the similar insecticidal activity against *H. cunea*.

The structure and function of 3D Cry toxin (Domain I/II/III) and the action mechanism of related target pests are relatively clear, which creates extremely favorable conditions for the functional transformation, especially the rational design of the improvement of insecticidal activity. Through site-directed mutagenesis of key functional sites in Domain I [26,49], targeted mutations in critical regions of the loops within Domain II [50,51] and modifications to the β-sheet regions of Domain III [28,52] were all performed to obtain mutants with improved insecticidal activity. Domain II and Domain III are responsible for the binding of Cry proteins to midgut receptors in target insects. Specifically, Domain II consists of three antiparallel β-sheets and is the most structurally variable region within the entire 3D structure of Cry proteins, exhibiting significant differences in length, charge, and conformation [53]. This variability is highly correlated with the specificity of insecticidal activity against different target pests and may influence the recognition of toxins by midgut receptors in target pests [54]. Studies have shown that Cry1Aa binds to its receptor through Loop a8, the β4a-β4b region, and Loop3 in Domain II, confirming the involvement of Domain II in receptor binding. Cry1Aa protein can bind to the ATP-binding cassette transporter (ABCC2) via Loop2 in Domain II [55]. The Loop2 and Loop3 of Cry1Ah interact with the binding region of APN1, a receptor in *Helicoverpa armigera* [27]. The three exposed loops in Domain II of Cry3Aa, spanning from amino acid residues 291 to 500, affect receptor affinity and toxicity [56]. The corresponding loop regions in Cry3Bb1 also play a crucial role in binding to insect receptors [57]. This study similarly found that mutants located on Loopα8 in Domain II enhanced toxicity of Cry9A against *H. cunea*. Compared with Cry9Aa3, the mutant protein 316LRG318AAA exhibits higher stability in the midgut juice of *H. cunea*, but there is no significant difference in its binding ability to the BBMVs of *H. cunea*. Previous research by Yang et al. also revealed that the Vip3A-S543N/I544L/S686R mutant protein exhibited 2.8-fold and 3.2-fold higher insecticidal activities against *Spodoptera frugiperda* and *H. armigera*, respectively, compared to the wild-type protein, which was also associated with improved stability in the midgut juice of these pests [58].

Both the Cry1Ea + Cry9Aa protein combination and the Cry1Ai + Cry9Aa protein combination exhibited significant synergistic effects against *Plutella xylostella* [48]. The combination of Cry9Aa3 and Vip3A proteins showed a synergistic effect against *C. suppressalis* [36]. Transgenic 741 poplar carrying a partially modified Bt gene and the Arrowhead Proteinase Inhibitor (API) gene demonstrated high resistance to Lepidopteran pests such as *Clostera anachoreta* and *L. dispar* [59]. In this study, we found that mixing Cry9Aa3 + Cry1Ah and 316LRG318AAA + Cry1Ah for bioassay revealed synergistic effects with synergistic factors of 4.76 and 8.33, respectively. The LC_50_ of the mutant combined with Cry1Ah protein against *H. cunea* was 0.48 μg/g, which is below 1 μg/g, indicating a promising application prospect. Furthermore, *cry9*-type genes, such as *cry9Ca* and *cry9Ee*, do not exhibit cross-resistance with *cry1Ac* or *cry1Ab* [38,39,60]. This combination of Cry9Aa3 and Cry1Ah could potentially delay the development of resistance compared to using a single Bt protein.

## 4. Materials and Methods

### 4.1. Insect Populations

*H. cunea* larvae were provided by the Chinese Academy of Forestry and Beijing Forestry University and have been continuously reared in the laboratory.

### 4.2. Strains and Mutant Proteins

The strains were stored in 50% glycerol stocks and preserved at -80, and Cry1Ah [61], Cry9 proteins [62], Cry9A3 (a truncated protein consisting of 1-655 amino acids) [63], and their mutant proteins [40] were all constructed in our lab. The *E. coli* expression plasmid carrying the wild-type cry9Aa3 active fragment gene that encodes N-terminal 655 amino acids was previously described. PCR amplification of *cry9Aa* expression plasmid was conducted with 14 pairs of primers (Table S8 containing mutant site sequences (Table S9) using Phusion Plus PCR Master Mix (Thermo Fisher Scientific, Waltham, MA, USA). The plasmid pEB containing the cry9Aa mutated gene was transformed into the *E. coli* DH5α competent cells (Zoman Biotechnology, Beijing, China) [40].

### 4.3. Protein Expression and Purification

*Escherichia coli* strains containing cry9 and cry9Aa3 mutants were grown in 300 mL of LB medium. Cells were lysed by sonication for 10 min (40% power, 3 s pulse on, 5 s pulse off) and centrifuged at 8000 rcf for 15 min at 4 °C. Proteins were purified by nickel-affinity chromatography [36], and the protein purification system AKTA (ÄKTA™ avant 25, Cytiva, Wilmington, NC, USA) was used with a desalting column (HiPrep™ 26/10 Desalting, Cytiva, USA) for protein desalting.

### 4.4. Bioassay

Eight grams of artificial diet (artificial diets containing soy flour (200 g/L), wheat bran (100 g/L), yeast (90 g/L), and multivitamin (15 mL/L)) [64] was used and placed in a Petri dish, and then 800 μL of the test sample solution was added. The mixture was thoroughly mixed and evenly spread in 6 cm Petri dishes to ensure that the insects could feed adequately. The dish was left at room temperature for a period of time, depending on the moisture level of the feed, until there are no water droplets on the surface. Twenty neonate larvae were placed into each dish. Three replicates were performed for each treatment. Diet supplemented with 20 mmol/L Tris-HCl buffer was used as negative control. The dishes were placed in a 25 °C intelligent artificial climate chamber with a photoperiod of 16:8 and a humidity of 50%. The moisture level of the feed was observed daily and appropriate adjustments were made as needed. After 96 h of incubation, the number of dead and live insects was counted, and the mortality rate, corrected mortality, and median lethal concentration (LC_50_) were calculated.

The corrected mortality was calculated as follows:

Corrected mortality = [(Treatment mortality − Control mortality)/(100 − Control mortality)] × 100.

LC_50_ values were determined through probit analysis performed with IBM SPSS Statistics 27 (version 27.0; IBM Corp., Armonk, NY, USA). The bioassay was independently replicated three times, with LC_50_ values derived from a single representative experiment. The Chi-squared values were calculated by SPSS software, and the slope values of dose–response curve were fitted by GraphPad Prism (GraphPad Prism 9, Dotmatics, UK).

The synergistic toxicity factor was calculated by the Tabashnik formula [65], which is as follows:

$$\frac{1}{{\;\mathrm{Expected}\;\mathrm{LC}}_{50}}=\frac{\begin{array}{c} \;\mathrm{Percentage}\;\mathrm{of}\\ \;A\;\mathrm{protein} \end{array}}{{\mathrm{LC}}_{50}\;\mathrm{of}\;A\;\mathrm{protein}}+\frac{\begin{array}{c} \mathrm{Percentage}\;\mathrm{of}\\ B\;\mathrm{protein} \end{array}}{{\mathrm{LC}}_{50}\;\mathrm{of}\;B\;\mathrm{protein}}$$

The synergistic toxicity factor = expected LC_50_/observed LC_50_. It is generally believed that a toxicity ratio of expected LC_50_ to observed LC_50_ between 0.5 and 2.6 indicates an additive effect, a ratio greater than 2.6 indicates a synergistic effect, and a ratio less than 0.5 indicates an antagonistic effect [66].

### 4.5. Stability Analysis of Cry9Aa3 and Mutant Proteins

The midgut tissues of about 15 third-instar larvae of *H. cunea* were placed in a clean tube. Surface lymph fluid was removed by washing with 0.7% NaCl solution. The midgut juice was prepared by centrifugation (Eppendorf 5424R, Hamburg, Germany). The supernatant was collected as midgut juice, and protein concentration was detected using a Bradford assay kit (Solarbio, Beijing, China). The midgut juice was diluted to different concentrations, and Cry9Aa3 and mutant proteins (200 μg) were incubated with the midgut juice at a volume ratio of 10:1 at 37 °C for 1 h. SDS-PAGE was used to detect protein activation and Image J was used for quantitative analysis.

### 4.6. Preparation of BBMVs

The midgut of third-instar *H. cunea* was dissected, quickly frozen in liquid nitrogen, and stored at −80 °C. The extraction method for BBMVs followed the magnesium precipitation method [67], and the purity of the extracted BBMVs was determined by comparing the specific activity enrichment of APN in the BBMVs with that in the initial midgut homogenate.

### 4.7. Saturation Binding Assays of Cry9Aa Proteins to BBMVs

The ELISA method was as follows: 100 μL of standard saline (PBS) containing 1 μg of BBMVs was added to the reaction hole of a 96-well plate and incubated at 4 °C overnight. The plate was washed three times with PBS buffer. PBS (100 μL) containing 5% skim milk was added and incubated at 37 °C for 2 h, then the wells were washed three times with PBS buffer. Gradient dilutions of Cry9Aa3 and mutant protein samples (100 μL) were added to the hole containing BBMVs and incubated at 37 °C for 2 h. The wells were washed three times with PBS containing 0.1% Tween-20. PBST (100 μL) containing 1/10000 Streptavidin-Horseradish Peroxidase was added and incubated at 37 °C for 1 h. TMB (100 μL) was added to each well and incubated at 37 °C for 15 min, then 100 μL of 2 mol/L HCl was added to each well to stop the reaction. The values at an OD of 450 nm were read, the deviation of the data in each group was analyzed, and Sigma-Plot software v15.0 was used to calculate the affinity of Cry9Aa3 and mutant proteins for BBMVs, as well as the equilibrium dissociation constant (*Kd*).

## 5. Conclusions

Both Cry9Aa3 and Cry9Aa4 proteins exhibit high insecticidal activity against *H. cunea*. It was found that the triple amino acid mutant 316LRG318AAA of Cry9Aa3 enhanced its insecticidal activity against *H. cunea*, which is associated with the increased stability of the mutant protein in the midgut juice of *H. cunea*, but there is no significant change in binding capacity with BBMVs. This indicates that the 316–318 sites in Domain II of Cry9Aa3 play a crucial role in protein structural stability. The Cry9Aa3 mutant 316LRG318AAA exhibits a synergistic effect with Cry1Ah, providing a gene combination reservoir for the creation of a new generation of transgenic poplars resistant to *H. cunea*.

## Figures and Tables

**Table 1:** Preliminary screening for bioassay of *H. cunea*.

Proteins	Concentration (μg/g)	Corrected Mortality (Mean ± SD, %)	Proteins	Concentration (μg/g)	Corrected Mortality (Mean ± SD, %)
R313A	5	23.08 ± 7.25 d	307PIG309AAA	5	23.08 ± 7.25 ^d^
	10	64.61 ± 6.53 ^bc^		10	31.66 ± 2.37 ^d^
S314A	5	38.46 ± 0.00 ^bcd^	316LRG318AAA	5	76.92 ± 3.63 ^a^
	10	59.42 ± 7.88 ^bc^		10	100.00 ± 0.00 ^a^
R368A	5	45.15 ± 3.63 ^bcd^	364TDR3666AAA	5	25.64 ± 3.63 ^d^
	10	74.74 ± 6.71 ^b^		10	61.98 ± 4.25 ^bc^
V369A	5	33.33 ± 3.63 ^bcd^	393HTT365AAA	5	48.72 ± 7.25 ^b^
	10	67.11 ± 3.00 ^bc^		10	74.61 ± 7.61 ^b^
R403A	5	41.03 ± 3.63 bcd	416NDT418AAA	5	20.51 ± 3.63 ^d^
	10	74.61 ± 7.61 ^b^		10	49.29 ± 8.06 ^c^
S498A	5	41.03 ± 3.63 ^bcd^	422VNR424AAA	5	41.03 ± 3.63 ^bcd^
	10	72.11 ± 4.07 ^b^		10	77.18 ± 3.99 ^b^
R569A	5	28.21 ± 7.25 ^cd^	Cry9Aa3	5	51.28 ± 3.63 ^b^
	10	64.48 ± 7.79 ^bc^		10	77.24 ± 3.18 ^b^
Q571A	5	39.46 ± 7.25 ^bdc^	-		
	10	74.74 ± 6.71 ^b^			

Different letters indicate significant difference using one-way analysis of variance (ANOVA), followed by LSD’s post hoc test. The original data were presented in Table S4.

**Table 2:** Insecticidal activity of Cry9Aa3 and 316LRG318AAA against *H. cunea*.

Proteins	Observed LC_50_ (μg/g)	Chi-Squared	Slope	Expected LC_50_ (μg/g)	Synergistic Factor
Cry9Aa3	5.48 (4.86–6.13)	11.19	1.68	-	-
316LRG318AA	3.69 (3.21–4.13)	8.96	1.34	-	-
Cry1Ah	4.36 (3.42–5.42)	11.23	0.97	-	-
Cry9Aa3 + Cry1Ah	1.02 (0.68–1.45)	5.54	0.85	4.76	4.76
316LRG318AA + Cry1Ah	0.48 (0.01–0.84)	8.02	1.32	4.00	8.33

The original data were presented in Table S7.

## Data Availability

All the original data were provided in Table S2–S7.

## Supplementary Materials

The following supporting information can be downloaded at: https://www.mdpi.com/article/10.3390/ijms26083497/s1.
