# Supplementary material for: Synergistic Insecticidal Activity Against Hyphantria cunea by Cry9Aa3 Mutants and Cry1Ah Combinations
Source: Int J Mol Sci. 2025 Apr 8;26(8):3497. doi: 10.3390/ijms26083497 (PMC12027373; doi:10.3390/ijms26083497)
Supplement: Supplementary file 1 [file ijms-26-03497-s001.zip › ijms-3505440-supplementary.pdf]

Supplementary materials

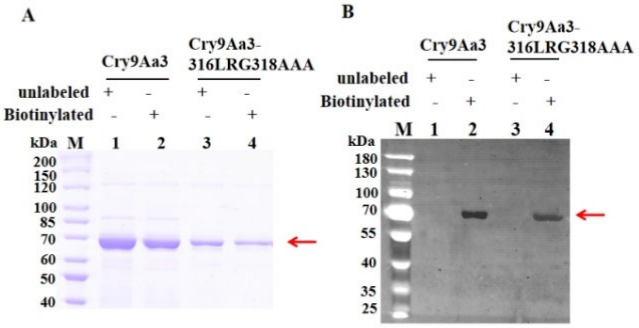

Figure S1. Biotinylation of Cry9Aa3 and its mutant proteins. (A) SDS-PAGE for detection of Cry9Aa3 and its mutant proteins before and after biotinylation. (B) Western-blot analysis of biotinylation of Cry9Aa3 and its mutant proteins.

Table S1. Statistics on Cry9-type proteins with insecticidal activity against Lepidopteran pest.

Protein Name	Target Species	Target Order
Cry9A	*Plutella xylostella, Helicoverpa armigera, Ostrinia furnacalis, Thaumatotibia leucotreta, Chilo suppressalis, Phthorimaea operculella, Epiphyas postvittana, Helicoverpa punctigera*	Lepidoptera
Cry9Ba	*Agrotis ipsilon, Helicoverpa zea*	Lepidoptera
Cry9Bb	*Spodoptera frugiperda, Manduca sexta, Anticarsia gemmatalis*	Lepidoptera
Cry9Ca	*Plutella xylostella, Lobesia botrana, Agrotis ipsilon, Choristoneura fumiferana, Epinotia aporema, Plutella xylostella, Cacyreus marshalli, Danaus plexippus, Plutella xylostella, Manduca sexta, Agrotis segetum, Heliothis virescens, Mamestra brassicae, Ostrinia nubilalis, Spodoptera exigua, Spodoptera littoralis*	Lepidoptera
Cry9Cb	*Plutella xylostella, Chilo suppressalis, Agrotis ipsilon, Ostrinia furnacalis, Mythimna separata*	Lepidoptera
Cry9Ea	*Cydia pomonella, Agrotis exclamationis*	Lepidoptera
Cry9Eb	*Ostrinia nubilalis, Plutella xylostella*	Lepidoptera
Cry9Ee	*Ostrinia nubilalis, Plutella xylostella, Plutella xylostella, Heliothis virescens, Ostrinia nubilalis*	Lepidoptera
Cry9Fa	*Ostrinia nubilalis*	Lepidoptera
Cry9Ga	*Plutella xylostella, Chrysodeixis includens*	Lepidoptera

Table S2. Bioassay of Cry9-Class proteins against larvae *Hyphantria cunea.*

Treatment	Total count			Survival count			Death count			Mortality (%)			Corrected mortality (%)		
	1	2	3	1	2	3	1	2	3	1	2	3	1	2	3
CK	20	20	20	20	20	19	0	0	1	0	0	0.05	/	/	/
Cry9Aa3	20	20	20	17	19	20	3	1	0	85	95	100	84.7	94.9	100
Cry9Aa4	20	20	20	20	20	20	0	0	0	100	100	100	100	100	100
Cry9Da4	20	20	20	3	3	4	17	17	16	15	15	20	13.6	13.6	18.5
Cry9Ee1	20	20	20	4	3	5	16	17	15	20	15	25	18.6	13.6	23.7
Cry9Eb2	20	20	20	5	3	5	15	17	15	25	15	25	23.7	13.6	23.7
Cry9Ee2	20	20	20	2	0	3	18	20	17	10	0	15	8.4	-1.69	23.7

Note: CK: 20 mM Tris-HCL.

Table S3. Bioassay of Cry9Aa3 and Cry9Aa4 proteins against larvae *H. cunea.*

Treatment	Concentration (μg/g)	Total count			Survival count			Death count			Mortality (%)			Corrected Mortality (%)		
		1	2	3	1	2	3	1	2	3	1	2	3	1	2	3
	0.5	20	20	20	20	19	19	0	1	1	0	0	0	0	0	0
	1	20	20	20	19	18	18	1	2	2	5	5	10	5	5	10
Cry9Aa3	2	20	20	20	16	17	15	4	3	5	20	5	30	20	5	30
	4	20	20	20	14	13	14	6	7	6	30	35	30	30	35	30
	8	20	20	20	5	4	7	15	16	13	75	70	65	75	70	65
	0.5	20	20	20	20	18	20	0	2	0	0	10	0	0	10	0
	1	20	20	20	19	19	19	1	1	1	5	0	10	5	0	10
Cry9Aa4	2	20	20	20	18	16	14	2	4	6	0	10	25	0	10	25
	4	20	20	20	12	18	17	8	2	3	40	10	15	40	10	15
	8	20	20	20	5	7	4	15	13	16	75	65	70	75	65	70
CK	/	20	20	20	20	20	20	0	0	0	0	0	0	/	/	/

Note: CK: 20 mM Tris-HCL.

Table S4. Bioassay of Cry9Aa3 and mutations against larvae *Hyphantria cunea.*

Treatment	Concentration (μg/g)	Total count		Survival count		Death count		Mortality (%)	
R313A	5	20	20	16	14	4	6	20	30
	10	20	20	9	6	11	14	55	70
S314A	5	20	20	12	12	8	8	40	40
	10	20	20	12	9	8	11	40	55
R368A	5	20	20	10	11	10	9	50	45
	10	20	20	6	7	14	13	70	65
V369A	5	20	20	14	12	6	8	30	40
	10	20	20	9	4	11	16	55	80
R403A	5	20	20	11	12	9	8	45	40
	10	20	20	3	7	17	13	85	65

S498A	5	20	20	11	12	9	8	45	40
	10	20	20	16	6	4	14	20	70
R569A	5	20	20	13	15	7	5	35	25
	10	20	20	15	9	5	11	25	55
Q571A	5	20	20	13	11	7	9	35	45
	10	20	20	10	14	10	6	50	30
307PIG309AAA	5	20	20	14	16	6	4	30	20
	10	20	20	14	16	6	4	30	20
316LRG318AAA	5	20	20	5	4	15	16	75	80
	10	20	20	9	1	11	19	55	95
364TDR3666AAA	5	20	20	15	14	5	6	25	30
	10	20	20	13	9	7	11	35	55
393HTT365AAA	5	20	20	11	9	9	11	45	55

continue

Treatment	Concentration (μg/g)	Total count		Survival count		Death count		Mortality (%)	
	10	20	20	4	6	16	14	80	70
416NDT418AAA	5	20	20	16	15	4	5	20	25
	10	20	20	4	5	16	15	80	75
422VNR424AAA	5	20	20	12	11	8	9	40	45
	10	20	20	4	5	16	15	80	75
Cry9Aa3	5	20	20	16	14	4	6	20	30
	10	20	20	9	6	11	14	55	70
CK		20	20	19	20	1	0	5	0

Note: CK: 20 mM Tris-HCL.

Table S5. Bioassay of Cry9Aa3 and Cry9Aa4 proteins against larvae *H. cunea.*

Treatment	Concentration	Total count			Survival count			Death count			Mortality (%)			Corrected Mortality (%)		
	(μg/g)	1	2	3	1	2	3	1	2	3	1	2	3	1	2	3
Cry9Aa3	0.5	20	20	20	20	19	18	0	1	2	0	5	10	-3.4	1.7	6.9
	1	20	20	20	18	18	16	2	2	4	10	10	20	6.9	6.9	17.2
	2	20	20	20	12	13	13	8	7	7	40	35	35	37.9	32.8	32.8
	4	20	20	20	10	9	7	10	11	13	50	55	65	48.3	53.4	63.8
	8	20	20	20	5	5	5	15	15	15	75	75	75	74.1	74.1	74.1
316LRG318AAA	0.5	20	20	20	18	17	17	2	3	3	10	15	15	6.9	12.1	12.1
	1	20	20	20	11	11	13	9	9	7	45	45	35	43.1	43.1	32.8
	2	20	20	20	10	9	10	10	11	10	50	55	50	48.3	53.4	48.3
	4	20	20	20	2	4	1	18	16	19	90	80	95	89.7	79.3	94.8
	8	20	20	20	0	0	0	20	20	20	100	100	100	100	100	100
CK	/	20	20	20	20	20	20	0	1	1	0	5	5	/	/	/

Note: CK: 20 mM Tris-HCL.

Table S6. Binding assay of Cry9Aa3 and 316LRG318AAA to BBMVs of *H. cunea*.

Protein (nM)	Cry9Aa3			316LRG318AAA		
400	2.63	2.76	2.70	2.76	2.81	2.79
200	2.56	2.61	2.52	2.61	2.60	2.61
100	2.15	2.08	2.14	2.45	2.48	2.47
50	1.22	1.31	1.22	1.47	1.50	1.55
25	0.63	0.64	0.64	0.74	0.77	0.77
12.5	0.32	0.28	0.28	0.43	0.36	0.38
6.25	0.15	0.14	0.12	0.19	0.17	0.22
CK	0.00	-0.01	0.00	-0.01	-0.01	0.03

Note: CK: PBS.

Table S7. LC_{50} bioassay against larvae *H. cunea.*

Treatment	Concentration (μg/g)	Total count			Survival count			Death count		
		1	2	3	1	2	3	1	2	3
Cry9Aa3	0.5	20	20	20	19	19	18	1	1	2
	1	20	20	20	18	17	17	2	3	3
	2	20	20	20	15	16	15	5	4	5
	4	20	20	20	11	12	12	9	8	8
	6	20	20	20	6	8	8	14	12	12
	8	20	20	20	4	5	6	16	15	14
	12	20	20	20	2	3	3	18	17	17
316LRG318AAA	0.5	20	20	20	18	18	18	2	2	2
	1	20	20	20	15	15	16	5	5	4
	2	20	20	20	13	13	15	7	7	5
	4	20	20	20	7	8	8	13	12	12
	6	20	20	20	4	4	6	16	16	14
	8	20	20	20	2	3	4	18	17	16
	12	20	20	20	0	0	0	20	20	20
3Cry1Ah	0.625	20	20	20	16	16	17	4	4	3
	1.25	20	20	20	13	14	14	7	6	6
	2.5	20	20	20	10	12	12	10	8	8
	5	20	20	20	5	7	10	15	13	10
	10	20	20	20	3	5	6	17	15	14
Cry9Aa3+Cry1Ah	0.15625	20	20	20	15	14	15	5	6	5
	0.3125	20	20	20	12	11	13	8	9	7
	0.625	20	20	20	11	10	11	9	10	9
	1.25	20	20	20	8	9	9	12	11	11
	2.5	20	20	20	5	5	5	15	15	15
	5	20	20	20	2	2	2	18	18	18
	10	20	20	20	0	0	0	20	20	20

316LRG318AAA +Cry1Ah	0.15625	20	20	20	13	12	12	7	8	8
	0.3125	20	20	20	11	11	11	9	9	9
	0.625	20	20	20	9	10	8	11	10	12
	1.25	20	20	20	6	7	5	14	13	15
	2.5	20	20	20	4	5	3	16	15	17
	5	20	20	20	2	1	0	18	19	20
	10	20	20	20	0	0	0	20	20	20
CK		20	20	20	20	20	19	0	0	1

Note: CK: 20 mM Tris-HCL.

Table S8. Information of primes

Primers	DNA sequences
307PIG309AAA	gggtcattatacagatgcagctgctttgtacatcgtagtagtcttagggg
316LRH318AAA	gttgtacatcgtagtagtgctgcggcagaaagttggtttagctttg
364TDR366AAA	cattgccggttagcccaagtgctgctgcagcgagggtatggtatggaagtcg
393HTT395AAA	ctgaactaatctctggacaagctgcggctgctacacaactatt
416NDT418AAA	gattctcaagcttgtaatttagctgctgccacatatggagtgaatagggcgg
422VNR424AAA	gtatttaaatgataccacatatggagcggctgcggcggtattatcatgatgcg
R313A	gatccaattgtttgtacatgctagtagtcttaggggagaaag
S314A	ccaattggtttgtacatcgtgctagtcttaggggagaaagttgg
R368A	gcccaagtactgatagagcggcggtatggtatggaagtcgagatc
V369A	caagtactgatagagcgagggcatggtatggaagtcgagatcg
R403A	ctacacaactattagggcaatatattagagtagattc
S498A	gtagcatctaatcgccgttcagctttagtaatgtatggttggac
R569A	ccattcacttcacttagcacaacaatatcgtattagagtc
Q571A	cattccacttcacttaagacaagcatatcgtattagagtccgttatgc

Table S9. Amino acid and base mutant of mutant proteins

Mutant protein name	Amino acid site	Primitive amino acid	Mutant Amino acids	Primitive base	Mutant base
307PIG309AAA	307-309	PIG	AAA	CCAATTGGT	GCAGCTGCT
316LRG318AAA	316-318	LRG	AAA	CTTAGGGGA	GCTGCGGCA
364TDR366AAA	364-366	TDR	AAA	ACTGATAGA	GCTGCTGCA
393HTT395AAA	393-395	HTT	AAA	CATACGACT	GCTGCGGCT
416NDT418AAA	416-418	NDT	AAA	AATGATACC	GCTGCTGCC
422VNR424AAA	422-424	VNR	AAA	GTGAATAGG	GCGGCTGCG
R313A	313	R	A	CGT	GCT
S314A	314	S	A	AGT	GCT
R368A	368	R	A	AGG	GCG
V369A	369	V	A	GTA	GCA
R403A	403	R	A	CGA	GCA

S498A	498	S	A	TCT	GCT
R569A	569	R	A	AGA	GCA
Q571A	571	Q	A	CAA	GCA

Note: A: Alanine; R: Arginine; N: Asparagine; P: Proline; I: Isoleucine; G: Glycine; L: Leucine; T: Threonine; D: Asparagine; H: Histidine; V: Valine; S: Serine; Q: Glutamine.
